# The dynamics of cost and benefit representations by noradrenaline and dopamine neuronal activity, and their relation to goal-directed behavior

**DOI:** 10.1186/1471-2202-12-S1-P262

**Published:** 2011-07-18

**Authors:** Barry J Richmond, Sebastien Bouret, Sabrina Ravel

**Affiliations:** 1 Laboratory of Neuropsychology, NIMH/NIH, Bethesda, MD 20892, USA; 2 Team Motivation Brain and Behavior, Institute du Cerveau et de la Moelle, Paris 75013, France; 3 Laboratoire de Neurobiologie de la Cognition, Université de Provence, CNRS, 13331 Marseille, France

## 

Higher brain functions that rely on attention and reward seeking depend on the integrity of neuromodulatory systems using dopamine (DA) and noradrenaline (NA). Despite the importance for these neuromodulators, there have been no direct experimental comparisons of the selectivity of DA and NA neurons, so making statements about the differences in functionality are speculative. We have recorded activity from dopamine neurons of the substantia nigra pars compacta and noradraline neurons of the locus coeruleus in monkeys while the monkeys carried out a visually-cued reward schedule task.In this task, the monkeys perform schedules of 1, 2, or 3 red-to-green sequential color discrimination trials to earn a reward. A cue presented at the beginning of each trial shows how many trials must yet be completed to obtain a reward. Error rates increase in this simple task as the number of trials yet to be performed before a reward increase, even though the red-green discrimination is identical in every trial, indicating that the reward value is discounted as a function of trials remaining before reward (at type of temporal discounting). We also find lip movements that occur when cue appears and at bar release (bar release is the correct response to a red->green color change). The lipping at the cue occurs whenever a new schedule begins, and is not related to the number of trials to be carried out. At bar release, lipping is strongest in rewarded trials. We interpret the lipping as reflecting the value of the event at that instant. Both DA and NA neurons seem to reflect the lipping response, with responses occurring when the cue appears in first trials, and at the time of bar-release. The neuronal responses that occur when the cue appears are similar, with both DA and NA neurons showing activations in the first trial of a schedule. At bar release, however, the activity furthest from reward is largest for NA neurons, whereas for DA neurons the greatest activity occurs in the rewarded trials.

Our data lead us to suggest that both systems are simultaneously evaluating the subjective or perceived value of the current state, but in different ways. We suggest that the noraderaline system reflects the cost of the current behavior, the cost being higher when the reward is more distant, and the dopamine system reflects the current expected benefit. In a neuroeconomic framework, these two catecholamine systems, almost certainly evolved from a common ancestor, have diverged to represent costs and benefits, for NA and DA, respectively. We speculate that the interaction between these two neuromodulatory systems is critical to motivate value-based behavior, and intimate knowledge of the dynamic balance between them is required to model the motivation underlying higher reward seeking activity.

